# The many faces of COPD in real life: a longitudinal analysis of the NOVELTY cohort

**DOI:** 10.1183/23120541.00895-2023

**Published:** 2024-02-12

**Authors:** Alvar Agustí, Rod Hughes, Eleni Rapsomaki, Barry Make, Ricardo del Olmo, Alberto Papi, David Price, Laura Benton, Stefan Franzen, Jørgen Vestbo, Hana Mullerova

**Affiliations:** 1 University of Barcelona, Respiratory Institute – Clinic Barcelona, IDIBAPS, and CIBERES, Barcelona, Spain; 2 Research and Early Development, Respiratory and Immunology, AstraZeneca, Cambridge, UK; 3 National Jewish Health and University of Colorado Denver, Denver, CO, USA; 4 Diagnostic and Treatment Department, Hospital de Rehabilitaciόn Respiratoria “Maria Ferrer” and IDIM CR, Buenos Aires, Argentina; 5 University of Ferrara, Department of Translation Medicine, Ferrara, Italy; 6 Observational and Pragmatic Research Institute, Singapore and Centre of Academic Primary Care, Division of Applied Health Sciences, University of Aberdeen, Aberdeen, UK; 7 University of Manchester and Manchester University NHS Foundation Trust, Manchester, UK; 8 These authors contributed equally

## Abstract

**Background:**

The diagnosis of COPD requires the demonstration of non-fully reversible airflow limitation by spirometry in the appropriate clinical context. Yet, there are patients with symptoms and relevant exposures suggestive of COPD with either normal spirometry (pre-COPD) or preserved ratio but impaired spirometry (PRISm). Their prevalence, clinical characteristics and associated outcomes in a real-life setting are unclear.

**Methods:**

To investigate them, we studied 3183 patients diagnosed with COPD by their attending physician included in the NOVELTY study (clinicaltrials.gov identifier NCT02760329), a global, 3-year, observational, real-life cohort that included patients recruited from both primary and specialist care clinics in 18 countries.

**Results:**

We found that 1) approximately a quarter of patients diagnosed with (and treated for) COPD in real life did not fulfil the spirometric diagnostic criteria recommended by the Global Initiative for Chronic Obstructive Lung Disease (GOLD), and could be instead categorised as pre-COPD (13%) or PRISm (14%); 2) disease burden (symptoms and exacerbations) was highest in GOLD 3–4 patients (exacerbations per person-year (PPY) 0.82) and lower but similar in those in GOLD 1–2, pre-COPD and PRISm (exacerbations range 0.27–0.43 PPY); 3) lung function decline was highest in pre-COPD and GOLD 1–2, and much less pronounced in PRISm and GOLD 3-4; 4) PRISm and pre-COPD were not stable diagnostic categories and change substantially over time; and 5) all-cause mortality was highest in GOLD 3–4, lowest in pre-COPD, and intermediate and similar in GOLD 1–2 and PRISm.

**Conclusions:**

Patients diagnosed COPD in a real-life clinical setting present great diversity in symptom burden, progression and survival, warranting medical attention.

## Shareable abstract

**This study presents the prevalence, clinical characteristics and associated outcomes of 3183 patients diagnosed with COPD, pre-COPD or PRISm in a global, real-life practice setting in the NOVELTY cohort** https://bit.ly/41d9PPz

## Introduction

According to the Global Initiative for Chronic Obstructive Lung Disease (GOLD), the diagnosis of COPD requires the demonstration of non-fully reversible airflow limitation, as indicated by the presence of a post-bronchodilator forced expiratory volume in 1 s (FEV_1_)/forced vital capacity (FVC) ratio <0.70, in the appropriate clinical context [1]. However, in real-life there are patients without spirometric evidence of airflow limitation (*i.e.* FEV_1_/FVC ratio >0.70), but with symptoms and/or other functional or structural alterations named pre-COPD [2], and others with preserved ratio but impaired spirometry (PRISm) (FEV_1_/FVC >0.70 and FEV_1_ <80% predicted) [3, 4]; both pre-COPD and PRISm patients are at risk of progression to airflow limitation (*i.e.* COPD), exacerbations and death [3–6]. This realisation can open new windows of opportunity for their prevention, early diagnosis and treatment, hence preventing the occurrence of COPD and facilitating healthier ageing [7–9].

Previous studies in pre-COPD or PRISm patients have been conducted either in the general population [6, 10–18] or in clinical cohorts of COPD patients [5, 19–26], so their prevalence, characteristics, treatment and temporal evolution in a real-life healthcare practice setting are unclear. Here, we sought to investigate these many faces of COPD in the NOVELTY cohort [27, 28], a global, large, prospective, observational (3-year), study in patients with a physician diagnosis of COPD recruited from both primary and specialist care clinics, including many who would not usually be included in clinical trials with more strictly defined COPD.

## Methods

### Study design, patients and ethics

The design of the NOVELTY study (clinicaltrials.gov identifier NCT02760329) has been described in detail previously [27, 28]. Briefly, patients with a physician diagnosis of COPD and/or asthma (n=11 226) were enrolled prospectively between September 2015 and March 2017 by their attending physicians, which included primary care physicians, pulmonologists and allergists, in community and hospital outpatient settings across 271 sites in 18 countries [27]. Patients were aged ≥12 years, had not participated in a respiratory interventional trial within the previous 12 months, and were likely to complete 3 years of follow-up. The current analysis only included those with a physician-assigned diagnosis of COPD (without concomitant asthma) for whom valid spirometric data was available at recruitment (n=3183). These patients were stratified based on their post-bronchodilator spirometry values into the following four diagnostic categories. 1) COPD GOLD 1–2 (FEV_1_/FVC ratio <0.70 and FEV_1_ ≥50% to <80% pred) [1]; 2) COPD GOLD 3–4 (FEV_1_/FVC ratio <0.7 and FEV_1_ <50% pred) [1]; 3) pre-COPD: diagnosis of COPD with normal spirometry (FEV_1_/FVC ratio ≥0.70 and FEV_1_ ≥80% pred) [2]; or 4) PRISm: diagnosis of COPD with FEV_1_/FVC ratio ≥0.70 and FEV_1_ <80% pred [29, 30]. The NOVELTY study was approved by the institutional review boards of each participating institution, and all patients provided written informed consent [27].

### Measurements

As detailed elsewhere [27, 28], at recruitment and yearly during 3 years of follow-up, demographics, smoking history, comorbidities, medications, respiratory symptoms and health status (modified Medical Research Council (mMRC) questionnaire on breathlessness, St George's Respiratory Questionnaire (SGRQ) and Chronic Airways Assessment Test (CAAT)), and rate of moderate-to-severe exacerbations in the previous 12 months as reported by the attending physician were registered. Frequent productive cough was defined as cough and sputum production most or several days/weeks for the past 3 months and derived from the SGRQ (scoring ≥3 for both SGRQ questions), as described previously [31]. Pre- and post-bronchodilator spirometry values were recorded following international recommendations [32], and FEV_1_ reversibility was defined by a change ≥12% and ≥200 mL after the administration of salbutamol (400 μg pressurised metered-dose inhaler). Reference values were those of the Global Lung Function Initiative [33]. Biomarker assessment included fractional exhaled nitric oxide (*F*_ENO_) and blood cell counts.

### Statistical analysis

Results are presented as mean±sd or number and proportions (denominators excluding patients with missing data). p-values are displayed for descriptive purposes and are based on the Chi-squared test for the comparison of categorical variables and the one-way ANOVA or Kruskal–Wallis H-test for normal or non-normal continuous variables, respectively. Multivariable logistic regression analysis adjusted for age, sex, smoking status and (unless otherwise specified) body mass index (BMI) was used to identify individual clinical factors associated with pre-COPD *versus* PRISm. We used alluvial plots to illustrate potential changes of disease category at recruitment (pre-COPD, PRISm, GOLD 1–2 and GOLD 3–4) over time that included all patients with complete data at all time points (n=995). These same population (n=995) was used to estimate FEV_1_ changes during follow-up in these four groups. Kaplan–Meier curves were used to compare all-cause mortality during follow-up across diagnostic categories established at baseline in the entire study population (n=3183), and Cox proportional hazards models (unadjusted and adjusted for age and sex) were used to estimate hazard ratios for the association between diagnostic categories and mortality. All analyses were performed in R (version 4.1.0).

## Results

### Characteristics of patients at recruitment

**FIGURE 1:**
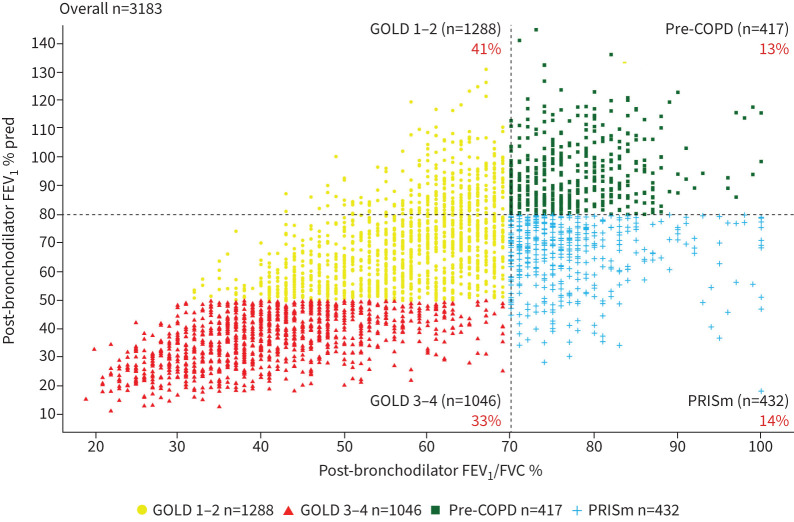
Scatter plot of post-bronchodilator forced expiratory volume in 1 s (FEV_1_)/forced vital capacity (FVC) (%) values *versus* post-bronchodilator FEV_1_ (% predicted) in patients with a physician-assigned diagnosis of COPD included in this analysis (n=3183). According to their measured spirometric values, patients are categorised as pre-COPD, preserved ratio but impaired spirometry (PRISm), Global Initiative for Chronic Obstructive Lung Disease (GOLD) 1–2 or GOLD 3–4. Proportions indicate the prevalence of each diagnostic category.

We included in this analysis 3183 patients diagnosed with COPD by their attending physician, mostly of Caucasian origin, many of them with a recent COPD diagnosis (47.5% diagnosed within the prior 5 years). As shown in figure 1, according to their spirometric values at recruitment, 417 (13%) had pre-COPD, 432 (14%) had PRISm, 1288 (41%) were GOLD 1–2 and 1046 (33%) GOLD 3–4.

Table 1 summarises the main demographic, clinical and biological characteristics of these four groups of patients. Pre-COPD and PRISm patients were ~5 years younger, included a larger proportion of females and had a higher BMI (particularly those with PRISm) than GOLD 1–2 or GOLD 3–4 patients. Their spirometric values are in line with their diagnostic criteria, defined in the methods section. The categorisation of patients in each of these four groups was similar when airway obstruction was defined using a fixed FEV_1_/FVC ratio <0.7 or its lower limit of normal (LLN). As shown in supplementary figure S1, reclassification was basically nonexistent in patients with pre-COPD, PRISm or GOLD 3–4. In patients with GOLD 1–2, 122 (9.5%) patients would have been reclassified to pre-COPD and 186 (14.4%) to PRISm if the LLN had been used instead. FEV_1_ reversibility was present in 11–15% of patients in each group (table 1). Pre-COPD and PRISm included a higher proportion of both never and current smokers, and cumulative smoking exposure (pack-years) was higher in GOLD 1–2 and GOLD 3–4 patients. Industrial and air pollution exposures were substantial and similar across groups. Symptom burden, as determined by mMRC, SGRQ, CAAT or frequent productive cough was higher in GOLD 3–4, but the respiratory symptom load and health status impairment was remarkable and similar in GOLD 1–2, pre-COPD and PRISm (table 1). A physician diagnosis of emphysema or bronchiectasis (sometimes, but not always, based on computed tomography imaging) was more frequent in patients with GOLD 1–2 or GOLD 3–4 than in those with pre-COPD or PRISm. Moderate–severe exacerbations of COPD in the year before recruitment were most frequent in GOLD 3–4 (47.8% with one or more events) but approximately a quarter of patients with GOLD 1–2, pre-COPD or PRISm reported 1 or more events according to the attending physician.

**TABLE 1:** Characteristics of participants with a physician diagnosis of COPD at recruitment in NOVELTY, by spirometric categories

	**GOLD 1–2**	**GOLD 3–4**	**Pre-COPD**	**PRISm**	**p-value^#^**
**Participants**	1288 (41)	1046 (33)	417 (13)	432 (14)	
**Spirometry**					
Post-bronchodilator FEV_1_/FVC %	57.2±8.3	41.0±10.3	77.9±6.1	77.3±7.0	0.142
Post-bronchodilator FEV_1_ % predicted	70.0±14.5	36.4±8.8	94.8±12.0	65.7±11.4	<0.001
Bronchodilator reversibility (≥200 mL and 12%)	17.7	10.4	9.6	13.3	0.091
**Demographics**					
Age years	67.7±9.0	67.6±8.2	62.8±11.5	63.3±10.1	0.476
Male	66.6	65.3	50.4	50.0	0.917
Body mass index kg·m^−2^	27.4±5.7	26.1±5.9	28.8±6.3	31.5±7.6	<0.001
**Exposures**					
Smoking status					0.534
Never	5.4	4.1	11.0	11.6	
Former	64.7	72.9	47.7	50.9	
Current	29.9	22.9	41.2	37.5	
Cumulative smoking exposure pack-years	44.7±37.4	50.5±45.4	33.4±33.5	33.4±32.1	0.972
Exposure to dust/fumes at work	41.9	44.2	44.9	41.8	0.387
**Symptoms**					
mMRC dyspnoea score ≥2	43.0	76.8	33.1	55.7	<0.001
SGRQ score	36.2±20.8	51.2±19.8	34.4±21.1	41.7±21.6	<0.001
CAAT total score [34]	15.4±8.0	19.9±7.8	14.6±7.9	17.7±8.6	<0.001
Frequent productive cough	34.8	43.9	29.4	35.9	0.110
Emphysema diagnosis^¶^	38.0	54.0	19.9	21.3	0.616
Bronchiectasis diagnosis^¶^	5.9	9.6	3.4	4.3	0.536
≥1 moderate–severe ECOPD previous year^¶^	29.4	47.8	22.1	25.3	0.275
**Comorbidities**					
Coronary artery disease	6.7	9.0	6.7	11.1	0.037
Type 2 diabetes	15.0	13.0	19.2	25.9	0.019
Rhinosinusitis	13.1	11.5	23.5	21.5	0.491
Gastro-oesophageal reflux	13.8	12.2	21.3	19.7	0.547
Depression/anxiety	13.0	14.6	18.7	24.1	0.057
**Biomarkers**					
*F*_ENO_ ppb	20.7±17.2	18.4±15.3	21.3±22.5	18.7±14.2	0.060
*F*_ENO_ excluding current smokers ppb	23.5±18.5	20.0±16.0	25.8±26.8	21.3±15.4	0.027
Blood eosinophils cells·µL^−1^	191.1±133.1	191.5±123.0	178.1±106.2	178.2±99.0	0.975
Blood neutrophils 10^9^ cells·L^−1^	4.7±1.7	5.4±1.9	4.6±1.8	4.9±2.0	0.202
**Medications (12 months before recruitment)**					
Reliever only (SABA, SAMA or both)	9.3	3.0	20.0	14.8	0.072
LAMA monotherapy	15.4	5.8	19.4	13.4	0.034
LABA monotherapy	2.5	0.6	0.6	2.8	0.029
LABA+ICS (without LAMA)	14.6	11.1	23.9	20.9	0.347
LABA+LAMA (without ICS)	23.4	18.5	11.8	14.8	0.250
LABA+LAMA+ICS	28.9	55.1	13.9	23.7	0.001
ICS (any combination)	48.8	70.9	47	53.1	0.110

Data are presented as n (%), mean±sd or %, unless otherwise stated. GOLD: Global Initiative for Chronic Obstructive Lung Disease; PRISm: preserved ratio but impaired spirometry; FEV_1_: forced expiratory volume in 1 s; FVC: forced vital capacity; mMRC: modified Medical Research Council; SGRQ: St George's Respiratory Questionnaire; CAAT: Chronic Airways Assessment Test; ECOPD: exacerbation of COPD; *F*_ENO_: fractional exhaled nitric oxide; SABA: short-acting β-agonist; SAMA: short-acting muscarinic antagonist; LAMA: long-acting muscarinic antagonist; LABA: long-acting β-agonist; ICS: inhaled corticosteroid. ^#^: p-values are shown for the pairwise comparison of PRISm *versus* pre-COPD (which are the focus of this article); all differences across the four groups were statistically significant at the ≤0.01 level, except for environmental exposures and blood eosinophils. ^¶^: as reported by the attending physician (often, but not always, based on computed tomography scan where relevant).

Comorbidities, including chronic heart disease (prior myocardial infarction or congestive heart failure), type 2 diabetes, rhinosinusitis, gastro-oesophageal reflux and depression/anxiety were more prevalent in pre-COPD and PRISm than in those with GOLD grades 1–2 or GOLD 3–4 (table 1). *F*_ENO_ values were similar across groups and about a quarter of patients in all groups had values >25 ppb. Blood eosinophil levels tended to be higher in GOLD 1–2 and GOLD 3–4, but blood neutrophils were similar in all groups (table 1). Finally, table 1 presents the medications used by these patients in the 12 months before recruitment. Of note, most patients with pre-COPD or PRISm were treated with one or two long-acting bronchodilators, often in combination with inhaled corticosteroids. All differences across the four groups were statistically significant at the ≤0.01 level, except for environmental exposures and blood eosinophils (table 1).

### Clinical factors associated with pre-COPD *versus* PRISm at recruitment

Multivariable logistic regression analysis adjusted for age, sex and smoking status showed that, compared to pre-COPD, PRISm was significantly associated with the following variables at baseline (supplementary figures S2–S4): obesity, more breathlessness (but not other respiratory symptoms) from a younger age, more frequent exacerbations and hospital admissions and some comorbidities, including type 2 diabetes and chronic heart disease; however, after adjusting for BMI, the association with these comorbidities disappear, suggesting a potentially relevant role of obesity. As a result, COPD was reported by the attending physician as being more severe in patients with PRISm than in patients with pre-COPD.

### Observations during follow-up

**FIGURE 2:**
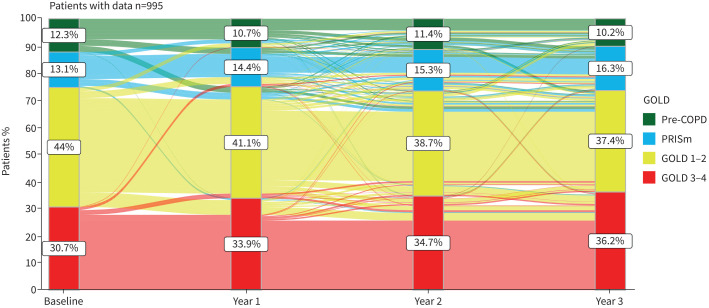
Alluvial plot of annual changes of the four diagnostic categories analysed here during follow-up, which includes all patients with complete data at all time points (n=995) and sets the minimum group change to 10 patients for visual clarity. Proportions denote the percentage of patients in each group. PRISm: preserved ratio but impaired spirometry; GOLD: Global Initiative for Chronic Obstructive Lung Disease.

Supplementary table S1 shows the proportion of patients who remained in (or changed) their baseline diagnostic category for 3 years of follow-up. Approximately three-quarters of GOLD 1–2 remained in the same diagnostic category over time, but 13–15% deteriorated and became GOLD 3–4, whereas 5–10% of them moved to pre-COPD or PRISm. In contrast, 90% of GOLD 3–4 were stable over time and only a few (10%) changed to either GOLD 1–2, pre-COPD or PRISm. Diagnostic variability over time in patients labelled at baseline as pre-COPD or PRISm was much larger, with only about 65% of them remaining in the same initial diagnostic category over time (supplementary table S1). These changes are illustrated graphically as an alluvial plot in figure 2, which only includes patients with complete data at all time points (n=995). The characteristics of this subgroup of patients at recruitment were not substantially different from the rest of the population studied (supplementary table S2). By and large, the four groups shown in figure 2 are stable over time, but there is the possibility of individual plasticity, particularly among pre-COPD and PRISm patients.

**FIGURE 3:**
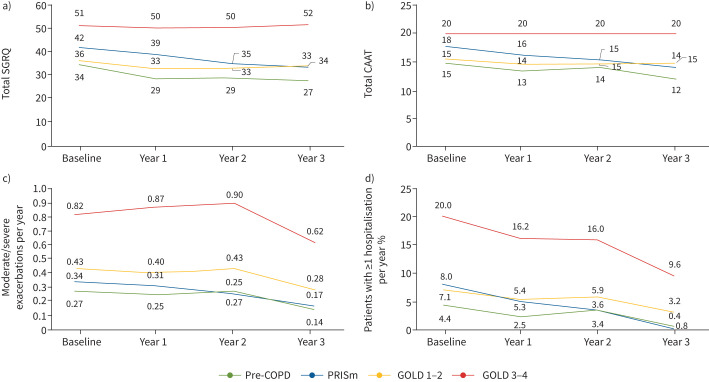
Mean values of health status as assessed by a) total St George's Respiratory Questionnaire (SGRQ) score or b) total Chronic Airways Assessment Test (CAAT) score; c) moderate/severe annual exacerbations; and d) proportion of patients with more than one hospitalisation because of a COPD exacerbation per year in patients with pre-COPD, preserved ratio but impaired spirometry (PRISm), Global Initiative for Chronic Obstructive Lung Disease (GOLD) 1–2 or GOLD 3–4, at baseline and each year during the 3-year follow-up. This graph includes all patients with data per visit, irrespective of whether they have data at other visits.

Figure 3 shows that symptom burden (figure 3a and b), exacerbation rate (figure 3c) and exacerbation severity (figure 3d) were highest in GOLD 3–4 patients, as expected. However, it is of note that they were similar in GOLD 1–2, pre-COPD and PRISm patients. By and large, there was a tendency towards decrease of these four variables during follow-up in all four groups. This is probably due to attrition of the study population over time (this graph includes all patients with data per visit, irrespective of whether they have data at other visits) and the well-described effect of the coronavirus disease 2019 pandemic on exacerbations rate between year 2 and year 3 [35, 36].

**FIGURE 4:**
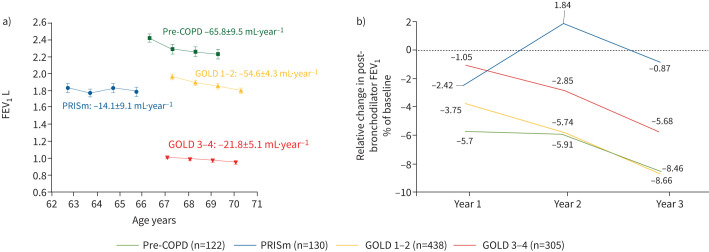
a) Mean±sem of forced expiratory volume in 1 s (FEV_1_) values determined at baseline and during follow-up (3 years) *versus* mean age (years) in patients with pre-COPD, preserved ratio but impaired spirometry (PRISm), Global Initiative for Chronic Obstructive Lung Disease (GOLD) 1–2 and GOLD 3–4 with valid data in all four visits (n=995). The mean±sem rate of absolute annual FEV_1_ decline is shown for each group. b) FEV_1_ values in each group at years (Y)1, 2 and 3 of follow-up, expressed as a percentage of the baseline (BSL) value (calculated as 100×(FEV_1(Y1, Y2, Y3)_ – FEV_1BSL_)/FEV1_BSL_).

Figure 4a shows that the annual FEV_1_ change was highest in pre-COPD (−65.8±9.5 mL·year^−1^) and GOLD 1–2 (−54.6±4.3 mL·year^−1^), and considerably less in GOLD 3–4 (−21.8±5.1 mL·year^−1^) and PRISm (−14.1±9.1 mL·year^−1^). Figure 4b shows the change in FEV_1_ during follow-up in the four groups of patients studied, expressed as percentage change from the baseline value, and confirms that PRISm and GOLD 3–4 patients had the lower change over time and that it was higher and similar in pre-COPD and GOLD 1–2. This is unlikely to be explained by changes in smoking status, because they were minor and similar across groups over time.

**FIGURE 5:**
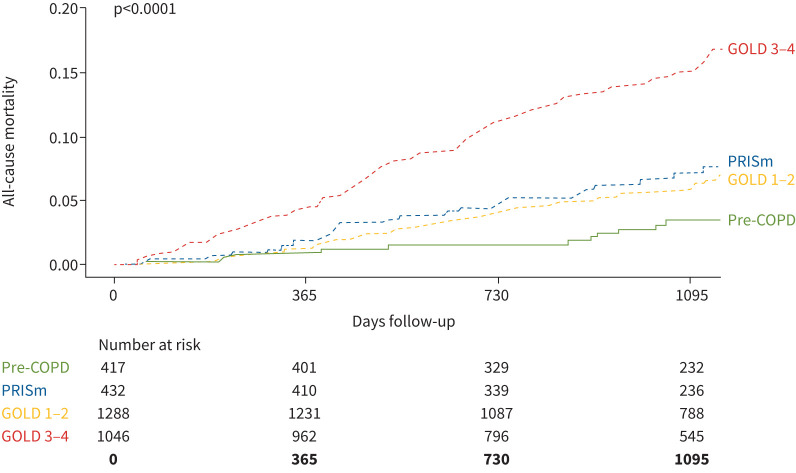
All-cause mortality during 3-year follow-up in pre-COPD, preserved ratio but impaired spirometry (PRISm), Global Initiative for Chronic Obstructive Lung Disease (GOLD) 1–2 and GOLD 3–4 patients.

Finally, figure 5 shows that all-cause mortality during follow-up was highest in GOLD 3–4, lowest in pre-COPD and intermediate and similar in GOLD 1–2 and PRISm patients. These comparisons were similar after adjustment for age and sex (supplementary table S3).

## Discussion

This study shows that 1) approximately a quarter (27%) of patients diagnosed with (and treated for) COPD in real-life primary and specialised care clinics do not actually have evidence of post-bronchodilator airflow limitation (the diagnostic COPD criteria recommended by GOLD [1]) and can instead be categorised as pre-COPD [2] (13%) or PRISm [3, 4] (14%); 2) as expected, disease burden (symptoms and exacerbations) was highest in GOLD 3–4 but, of note, it was similar in pre-COPD, PRISm and GOLD 1–2 patients; 3) membership of the pre-COPD and PRISm diagnostic categories established at recruitment is dynamic and can change over time (3-year follow-up); 4) FEV_1_ decline was abnormally accelerated in pre-COPD and GOLD 1–2, but normal in PRISm and GOLD 3–4; and 5) all-cause mortality was highest in GOLD 3–4, lowest in pre-COPD, and intermediate and similar in GOLD 1–2 and PRISm. Collectively, these observations provide a unique picture of the many faces of COPD in a real healthcare setting.

### Previous studies

Several previous studies have investigated the characteristics and clinical relevance of pre-COPD and PRISm patients in the general population [6, 10–18] or in clinical cohorts of COPD patients [5, 19–26]. To our knowledge, this is the first to investigate them in a global, large, prospective healthcare practice setting that include patients with a physician diagnosis of COPD recruited from both primary and specialist care clinics [27, 28].

### Interpretation of findings and clinical implications

Our understanding of COPD has changed significantly over the past few years. Traditionally, COPD was considered a self-inflicted disease by tobacco smoking occurring in older males [37]. Now we know that other risk factors besides smoking also increase the risk of COPD [38–40]; that the disease similarly affects males and females [41]; that different lung function trajectories through the lifetime involving abnormal lung development and/or accelerated ageing can lead to COPD [40, 42–44]; and that, as a result, the disease can also be identified in young adults [18]. The results of this study contribute to better delineate some of these many faces of COPD in a real-life setting.

First, 27% of patients diagnosed with COPD in primary and specialised care did not exhibit the diagnostic criteria proposed by GOLD [1]. Instead, 13% had a normal spirometry (pre-COPD) and 14% PRISm (figure 1). These figures are in the range of those reported previously in the general population [6, 10–18] or COPD patient cohorts [5, 19–24]. It is possible that other potential comorbidities (*e.g.* chronic heart failure/diastolic dysfunction) may mimic the symptoms of COPD and lead to an incorrect diagnosis; unfortunately, NOVELTY does not include any echocardiographic measurement that allow us to explore this possibility, albeit it may be of note that PRISm patients had a higher reported incidence of coronary artery disease (table 1). Conversely, it may be informative to explore how these two diagnostic categories without airflow obstruction (pre-COPD and PRISm) compare to canonical COPD with airflow obstruction. In this context, it is of note that pre-COPD and PRISm patients were younger, included more females, and, interestingly, a higher proportion of both never-smokers and current smokers than COPD (table 1). Nonetheless, disease burden (symptoms, exacerbations) in both groups was similar to that of GOLD 1–2 patients and most of them were treated with one or two bronchodilators, often in combination with inhaled corticosteroids, like COPD (table 1). This is in keeping with previous observations in the SPIROMICS study [45] and clearly illustrates the need for well-designed clinical trials in these patients [8, 24]. Recent evidence has shown that inhaled dual bronchodilator therapy does not decrease respiratory symptoms in symptomatic, tobacco-exposed persons with normal spirometry in the short term (12 weeks) [24]. Clearly, longer studies exploring the potential impact of this or other treatment options in other clinically relevant outcomes such as FEV_1_ decline (figure 3), rate of exacerbations (figure 4) and/or long-term mortality (figure 5) in pre-COPD and PRISm patients are needed [8]. In the meantime, careful monitoring and *lex artis* therapeutic management is advised in pre-COPD and PRISm patients through the search and treatment of potential treatable traits in each individual patient [46, 47].

Second, another clinically relevant question is to what extent the pre-COPD and PRISm diagnostic categories describe similar or different subpopulations of patients. Our results show that the proportion of females and smoking exposure was similar in both groups, but PRISm patients were more obese, more symptomatic from a younger age, and tended to suffer more comorbidities, exacerbations and hospital admissions, as other studies have shown [4]; as a result, their disease may have been considered more severe by the attending physician. Besides, pre-COPD and PRISm patients followed very different FEV_1_ trajectories during follow-up. Whereas the former exhibited accelerated FEV_1_ decline over 3 years [48], similar to that of GOLD 1–2, PRISm patients remained essentially stable during follow-up (figure 3), in keeping with observations in the general population in the Rotterdam Study [10] and in the COPDGene cohort [5]. Finally, mortality was lowest in pre-COPD but higher and similar in PRISm and GOLD 1–2 (figure 4). Collectively, therefore, these differences support that pre-COPD and PRISm identify different subpopulations of patients. That PRISm patients are more obese, more symptomatic and comorbid, likely not to present accelerated lung function decline with time, but at a higher risk of mortality than pre-COPD, is compatible with previous reports in the Framingham Offspring Cohort showing that patients with reduced lung function in early adulthood have a higher incidence of premature comorbidities and early death [49]. These observations are compatible with the hypothesis that PRISm individuals may have not developed their lungs (and perhaps other organ systems too (multimorbidity)) properly during infancy and adolescence, and may therefore represent a distinct population from pre-COPD patients [50].

Third, the longitudinal design of our study allowed us to investigate the stability of the pre-COPD, PRISm, GOLD 1–2 and GOLD 3–4 diagnostic categories over time. As illustrated graphically in the alluvial plot shown in figure 2 (and numerically in supplementary table S1) we found that during the 3-year follow-up most COPD patients (particularly GOLD 3–4) remained in the same diagnostic category, whereas only about 65% of pre-COPD and PRISm patients remaining stable in the same initial diagnostic category over time, the rest deteriorating or improving in roughly the same proportion. This temporal variability is in keeping with previous studies [5, 10, 15, 20, 51–53].

Finally, some observations in relation to FEV_1_ decline over time are worth discussing. First, as already known [1], FEV_1_ decline was higher in GOLD 1–2 than GOLD 3–4 (both in absolute values and when expressed as percentage of the baseline value; figure 4a and b). This is in keeping with the well-known “horse-racing effect” which describes the fact that those with more preserved lung function have more to lose and thus may have larger absolute decline [54]. Of note, all-cause mortality was much higher in GOLD 3–4 (figure 5). This may be in keeping with previous observations reporting that individuals with reduced peak lung function in early adulthood have reduced FEV_1_ decline [42], but increased mortality later in life [49]. And second, FEV_1_ decline was highest in pre-COPD patients (figure 4). This is compatible with these individuals belonging to a supranormal lung function trajectory characterised by accelerated lung function decline starting from a supranormal peak lung function in early adulthood [44, 55, 56], supporting again the horse-racing effect discussed earlier [54]. Whether or not patients with pre-COPD or PRISm in the NOVELTY cohort will eventually develop airflow obstruction (*i.e.* COPD) cannot be ascertained from our observations. We know from other studies that not all PRISm or pre-COPD patients will do so [1]. We speculate that, given that the patients studied here are in their sixties, the development of severe COPD is unlikely. However, a recent analysis of the SPIROMICS cohort showed that symptomatic smokers with normal spirometry do not have accelerated rates of FEV_1_ decline or increased incidence of COPD *versus* those with without symptoms, but experience more exacerbations during follow-up [26]. In any case, collectively, our observations clearly support that it is possible to identify different lung function trajectories in a real-life setting that are probably associated to different mechanisms [42–44], biomarkers [57] and outcomes (figure 5). However, a word of caution is necessary here since the observations on FEV_1_ decline (figure 3) are based on a relatively small (n=995) fraction of patients (*i.e.* those who with valid spirometry at all four points), and some of them may not have linear deterioration, or may even improve, as the alluvial plot indicates (figure 2).

### Strengths and limitations

Our study has several strengths and limitations. Among the former, 1) while several of these observations have been reported in other cohorts, a clear strength of these findings is an unselected global population of subjects diagnosed with COPD, thereby supporting similar findings in selected cohorts (COPDGene [25], SPIROMICS [26, 58]) in a more general population; 2) these data support the evolving concepts in the diagnosis of COPD, showing that a traditional spirometric cut-off of an FEV_1_/FVC <0.70 to support the diagnosis of COPD may not be necessary to diagnose (or rule out) COPD [59]; and 3) this analysis provides novel and relevant data on the longitudinal stability of these diagnostic categories as well as about their relationship with clinically relevant outcomes, which has not been precisely defined by previous studies in other general population [6, 10–18] or clinical cohorts of patients with COPD [5, 19–24].

Among potential limitations we acknowledge that, first, the criteria to determine a diagnosis of COPD used by physicians caring for these patients may potentially not conform to current guidelines [1]. For instance, we used post-bronchodilator spirometric values for the diagnosis of COPD [1] and we excluded patients with a diagnosis of asthma. However, we admit that in real life there are patients diagnosed with “asthma” who may actually have COPD, and that post-bronchodilator (or even pre-bronchodilator) spirometry is often not measured. Second, the study population was not a random sample of the general population, as there were target numbers for recruitment by diagnosis and severity to ensure adequate samples for subgroup analyses, there was patient dropout during follow-up and findings of this analysis represent the characteristics of patients already on treatment, which may differ from those at the time of initial diagnosis; in addition, our mortality findings are short-term (3 years) and we do not have specific information on cause(s) of death; and finally, we cannot exclude a healthy survivor effect in the analysis of longitudinal data since the analysis of change of lung function over time used completers only, as it would not be possible to compare across time points if different patients were contributing to each time point. Third, NOVELTY lacks imaging data to better understand the potential structural alterations of pre-COPD and PRISm patients. Fourth, because there was considerable attrition (69%) over the follow-up period, the analysis of the longitudinal FEV_1_ changes (figure 4) was restricted to those with complete follow-up data at all four time points (n=995); this may have led to some selection bias, albeit most baseline characteristics were similar (supplementary table S2) in those with incomplete data (n=2188) *versus* those with complete follow-up data (n=995). Furthermore, because we studied patients in their sixties and follow-up was only 3 years, the proposal that pre-COPD and PRISM patients are actually members of certain lung function trajectories is speculative. Finally, despite that NOVELTY also includes patients diagnosed by their attending physicians of asthma or asthma and COPD [28], in this analysis we focused exclusively on those diagnosed by their attending COPD physician to reduce the heterogeneity of the population studied.

### Conclusions

This analysis shows that approximately a quarter of patients diagnosed and treated as COPD in real life do not fulfil the COPD definition of non-fully reversible airflow limitation and can be classified instead as pre-COPD or PRISm; yet their disease burden and temporal progression is similar to that of those with spirometrically confirmed GOLD 1–2 COPD. These patients deserve careful attention and eventual treatment, particularly those with pre-COPD, who lose lung function at a very high rate. Well-designed, randomised clinical trials are urgently needed to determine the best therapeutic options for all these patient types [8].

## Supplementary material

10.1183/23120541.00895-2023.Supp1 **Please note:** supplementary material is not edited by the Editorial Office, and is uploaded as it has been supplied by the author.
Supplementary material 00895-2023.SUPPLEMENT
Figure S1 00895-2023.FIGURES1
Figure S2 00895-2023.FIGURES2
Figure S3a 00895-2023.FIGURES3A
Figure S3b 00895-2023.FIGURES3B
Figure S3c 00895-2023.FIGURES3C
Figure S4 00895-2023.FIGURES4
