# Supplementary material for: The many faces of COPD in real life: a longitudinal analysis of the NOVELTY cohort
Source: ERJ Open Res. 2024 Feb 12;10(1):00895-2023. doi: 10.1183/23120541.00895-2023 (PMC10860203; doi:10.1183/23120541.00895-2023)
Supplement: Supplementary file 1 [file 00895-2023.SUPPLEMENT.pdf]

27 November 2023

European Respiratory Journal Open Research

ON-LINE SUPPLEMENT

THE MANY FACES OF COPD IN REAL LIFE:
A LONGITUDINAL ANALYSIS OF THE NOVELTY COHORT

Alvar Agusti[1*], Rod Hughes[2*], Eleni Rapsomaki[2], Barry Make[3], Ricardo del Olmo[4], Alberto Papi[5], David Price[6], Laura Benton[2], Stefan Franzen[2], Jørgen Vestbo[7], and Hana Mullerova[2] for the NOVELTY Scientific Community and the NOVELTY study investigators

*co-primary authors

[1] University of Barcelona, Respiratory Institute - Clinic Barcelona, IDIBAPS, and CIBERES, Barcelona, Spain

[2] Research and Early Development, Respiratory & Immunology, AstraZeneca, Cambridge, UK

[3] National Jewish Health and University of Colorado Denver, Denver, CO, USA

[4] Diagnostic and Treatment Department, Hospital de Rehabilitación Respiratoria "Maria Ferrer" and IDIM CR, Buenos Aires, Argentina

[5] University of Ferrara, Department of Translation Medicine, Ferrara, Italy

[6] Observational and Pragmatic Research Institute, Singapore and Centre of Academic Primary Care, Division of Applied Health Sciences, University of Aberdeen, Aberdeen, UK

[7] University of Manchester and Manchester University NHS Foundation Trust, Manchester, UK

Correspondence: Dr. Alvar Agustí, Institut Respiratori, Clinic Barcelona, C/Villarroel 170, 08036 Barcelona, 08036, Spain. Tel: +34 93 2271701; E-mail: aagusti@clinic.cataagusti@clinic.cat

Running header*:* COPD faces in real-life

Financial Support: The NOVELTY study is funded by AstraZeneca. The sponsor participated in the design of the study, data analysis, and preparation of the manuscript.

On-line supplement: Tables: 5; Figures: 4

Table S1. Number (and proportion) of patients stratified by category group at recruitment (and proportion *vs.* baseline), at year 1, 2 and 3 of follow-up. Bold figures identify the proportion of patients remaining in the same initial diagnostic category over time. For further explanations, see text.

BASELINE	GOLD 1-2 (N=1288)	GOLD 3-4 (N=1046)	PRE-COPD (N=417)	PRISm (N=432)
**Year 1**				
GOLD 1-2	**776 / 1044 (74.3%)**	58 / 776 (7.5%)	40 / 304 (13.2%)	58 / 322 (18.0%)
GOLD 3-4	137 / 1044 (13.1%)	**688 / 776 (88.7%)**	3 / 304 (1.0%)	20 / 322 (6.2%)
Pre-COPD	49 / 1044 (4.7%)	6 / 776 (0.8%)	**210 / 304 (69.1%)**	39 / 322 (12.1%)
PRISm	82 / 1044 (7.9%)	24 / 776 (3.1%)	51 / 304 (16.8%)	**205 / 322 (63.7%)**
**Year 2**				
GOLD 1-2	**531 / 748 (71.0%)**	29 / 546 (5.3%)	41 / 225 (18.2%)	45 / 228 (19.7%)
GOLD 3-4	115 / 748 (15.4%)	**492 / 546 (90.1%)**	7 / 225 (3.1%)	15 / 228 (6.6%)
Pre-COPD	33 / 748 (4.4%)	3 / 546 (0.5%)	**144 / 225 (64.0%)**	29 / 228 (12.7%)
PRISm	69 / 748 (9.2%)	22 / 546 (4.0%)	33 / 225 (14.7%)	**139 / 228 (61.0%)**
**Year 3**				
GOLD 1-2	**371 / 535 (69.3%)**	24 / 351 (6.8%)	32 / 150 (21.3%)	28 / 161 (17.4%)
GOLD 3-4	99 / 535 (18.5%)	**313 / 351 (89.2%)**	3 / 150 (2.0%)	9 / 161 (5.6%)
Pre-COPD	28 / 535 (5.2%)	3 / 351 (0.9%)	**79 / 150 (52.7%)**	15 / 161 (9.3%)
PRISm	37 / 535 (6.9%)	11 / 351 (3.1%)	36 / 150 (24.0%)	**109 / 161 (67.7%)**

Table S2. Comparison of clinical characteristics between patients with complete data throughout baseline to year 3 vs patients with missing data.

	Patients with incomplete follow-up data (N=2188)	Patients with complete follow-up data (N=995)	Total (N=3183)	p value
**Spirometry**				
Post-BD FEV_1/FVC, %	57.6 (16.7)	56.8 (15.8)	57.3 (16.5)	0.208
Post-BD FEV_1 (% predicted)	60.9 (23.1)	63.1 (22.9)	61.6 (23.0)	0.014
Broncho-reversibility (≥200mL and 12%)	286 / 2070 (13.8%)	129 / 971 (13.3%)	415 / 3041 (13.6%)	0.691
**Demographics**				
Age, years	66.4 (9.9)	66.5 (8.4)	66.4 (9.5)	0.708
Males	1352 / 2188 (61.8%)	615 / 995 (61.8%)	1967 / 3183 (61.8%)	0.993
BMI kg/m^2	27.7 (6.5)	27.8 (6.2)	27.7 (6.4)	0.816
**Exposures**				
Smoking status				< 0.001
- Current smoker	722 / 2187 (33.0%)	237 / 995 (23.8%)	959 / 3182 (30.1%)	
- Former smoker	1323 / 2187 (60.5%)	692 / 995 (69.5%)	2015 / 3182 (63.3%)	
- Never smoked	142 / 2187 (6.5%)	66 / 995 (6.6%)	208 / 3182 (6.5%)	
Cumulative smoking exposure, pack years	43.3 (40.4)	44.3 (38.0)	43.6 (39.7)	0.535
Exposure to dust/fumes at work	899 / 2022 (44.5%)	373 / 935 (39.9%)	1272 / 2957 (43.0%)	0.020
**Symptoms**				
mMRC dyspnoea >=2	1231 / 2164 (56.9%)	482 / 982 (49.1%)	1713 / 3146 (54.5%)	< 0.001

	Patients with incomplete follow-up data (N=2188)	Patients with complete follow-up data (N=995)	Total (N=3183)	p value
SGRQ Total score	44.1 (22.0)	37.8 (20.8)	41.9 (21.8)	< 0.001
CAAT Total	17.8 (8.3)	15.8 (8.0)	17.1 (8.3)	< 0.001
Freq productive cough	561 / 1426 (39.3%)	266 / 784 (33.9%)	827 / 2210 (37.4%)	0.012
Emphysema diagnosis*	808 / 2188 (36.9%)	421 / 995 (42.3%)	1229 / 3183 (38.6%)	0.004
Bronchiectasis diagnosis*	136 / 2188 (6.2%)	72 / 995 (7.2%)	208 / 3183 (6.5%)	0.280
≥1 moderate-severe ECOPD previous year*	751 / 2174 (34.5%)	325 / 993 (32.7%)	1076 / 3167 (34.0%)	0.317
**Comorbidities**				
Coronary artery disease	184 / 2188 (8.4%)	72 / 995 (7.2%)	256 / 3183 (8.0%)	0.259
Type 2 diabetes	369 / 2188 (16.9%)	152 / 995 (15.3%)	521 / 3183 (16.4%)	0.262
Rhinosinusitis	324 / 2188 (14.8%)	156 / 995 (15.7%)	480 / 3183 (15.1%)	0.525
Gastro-oesophageal reflux	315 / 2188 (14.4%)	175 / 995 (17.6%)	490 / 3183 (15.4%)	0.021
Depression / anxiety	345 / 2188 (15.8%)	157 / 995 (15.8%)	502 / 3183 (15.8%)	0.994
**Biomarkers**				
Exhaled nitric oxide (FeNO), ppb	19.3 (16.3)	20.7 (18.6)	19.7 (17.1)	0.054
Expired nitric oxide excluding current smokers, ppb	22.0 (17.8)	22.8 (19.9)	22.3 (18.5)	0.383
Blood eosinophils/μL	184.5 (128.2)	179.6 (116.1)	182.7 (123.9)	0.441

	Patients with incomplete follow-up data (N=2188)	Patients with complete follow-up data (N=995)	Total (N=3183)	p value
Blood neutrophils (10^9/L)	5.0 (1.9)	4.8 (1.7)	4.9 (1.8)	0.045
**Treatment**				
Reliever only (SABA, SAMA or both)	195 / 1875 (10.4%)	57 / 897 (6.4%)	252 / 2772 (9.1%)	< 0.001
LAMA monotherapy	218 / 1875 (11.6%)	122 / 897 (13.6%)	340 / 2772 (12.3%)	0.138
LABA monotherapy	28 / 1875 (1.5%)	18 / 897 (2.0%)	46 / 2772 (1.7%)	0.322
LABA+ICS	327 / 1875 (17.4%)	97 / 897 (10.8%)	424 / 2772 (15.3%)	< 0.001
LABA+LAMA	323 / 1875 (17.2%)	210 / 897 (23.4%)	533 / 2772 (19.2%)	< 0.001
LABA+LAMA+ICS	660 / 1875 (35.2%)	326 / 897 (36.3%)	986 / 2772 (35.6%)	0.556
ICS (any combination)	1093 / 1875 (58.3%)	483 / 897 (53.8%)	1576 / 2772 (56.9%)	0.027

*as reported by the attending physician (often but not always based on CT scan)

Table S3. Hazard ratios (vs Pre-COPD) for the association between PRISm and GOLD stages 1-2 and 3-4 with mortality based on Cox PH models.

	**Unadjusted HR (95% CI)**	**Adjusted HR (95% CI)***
Pre-COPD	Reference	
PRISm	2.44 [1.25, 4.76]	2.53 [1.30, 4.95]
GOLD 1-2	2.19 [1.19, 4.01]	1.83 [1.00, 3.35]
GOLD 3-4	5.54 [3.08, 9.97]	4.70 [2.61, 8.46]

CI, Confidence interval/

* Covariates for adjustment were age and sex.

Table S4. List of NOVELTY Scientific Community members

Scientific Community member	Country	Scientific Community member	Country
Ricardo del Olmo	Argentina	Stefan Franzén	AstraZeneca, Sweden
Gary Anderson	Australia	Christina Keen	AstraZeneca, Sweden
Helen Reddel	Australia	Kristoffer Ostridge	AstraZeneca, Sweden
Marcelo Rabahi	Brazil	James Chalmers	UK
Andrew McIvor	Canada	Timothy Harrison	UK
Mohsen Sadatsafavi	Canada	Ian Pavord	UK
Ulla Weinreich	Denmark	David Price	UK
Pierre-Régis Burgel	France	Adnan Azim	AstraZeneca, UK
Gilles Devouassoux	France	Laura Belton	AstraZeneca, UK
Alberto Papi	Italy	Francois-Xavier Blé	AstraZeneca, UK
Hiromasa Inoue	Japan	Clement Erhard	AstraZeneca, UK
Adrián Rendon	Mexico	Kerry Gairy	AstraZeneca, UK
Maarten van den Berge	Netherlands	Rod Hughes	AstraZeneca, UK
Richard Beasley	New Zealand	Glenda Lassi	AstraZeneca, UK
Alvar Agusti García-Navarro	Spain	Hana Müllerová	AstraZeneca, UK
Rosa Faner	Spain	Eleni Rapsomaniki	AstraZeneca, UK
José Olaguibel Rivera	Spain	Ian Christopher Scott	AstraZeneca, UK
Christer Janson	Sweden	Bradley Chipps	USA
Magdalena Bilińska-Izydorczyk	AstraZeneca, Sweden	Stephanie Christenson	USA
Malin Fagerås	AstraZeneca, Sweden	Barry Make	USA
Titti Fihn-Wikander	AstraZeneca, Sweden	Erin Tomaszewski	AstraZeneca, USA

Table S5. List of NOVELTY study investigators

Investigator	Country	Investigator	Country
**Ricardo del Olmo**	**Argentina**	**Hiromasa Inoue**	**Japan**
Gabriel Benhabib	Argentina	Takeo Endo	Japan
Xavier Bocca Ruiz	Argentina	Masaki Fujita	Japan
Raul Eduardo Lisanti	Argentina	Yu Hara	Japan
Gustavo Marino	Argentina	Takahiko Horiguchi	Japan
Walter Mattarucco	Argentina	Keita Hosoi	Japan
Juan Nogueira	Argentina	Yumiko Ide	Japan
Maria Parody	Argentina	Minehiko Inomata	Japan
Pablo Pascale	Argentina	Koji Inoue	Japan
Pablo Rodriguez	Argentina	Sumito Inoue	Japan
Damian Silva	Argentina	Motokazu Kato	Japan
Graciela Svetliza	Argentina	Masayuki Kawasaki	Japan
Carlos F. Victorio	Argentina	Tomotaka Kawayama	Japan
Roxana Willigs Rolon	Argentina	Toshiyuki Kita	Japan
Anahi Yañez	Argentina	Kanako Kobayashi	Japan
**Helen Reddel**	**Australia**	Hiroshi Koto	Japan
Stuart Baines	Australia	Koichi Nishi	Japan
Simon Bowler	Australia	Junpei Saito	Japan
Peter Bremner	Australia	Yasuo Shimizu	Japan
Sheetal Bull	Australia	Toshihiro Shirai	Japan
Patrick Carroll	Australia	Naruhiko Sugihara	Japan
Mariam Chaalan	Australia	Ken-ichi Takahashi	Japan
Claude Farah	Australia	Hiroyuki Tashimo	Japan
Gary Hammerschlag	Australia	Keisuke Tomii	Japan
Kerry Hancock	Australia	Takashi Yamada	Japan
Zinta Harrington	Australia	Masaru Yanai	Japan
Gregory Katsoulotos	Australia	**Adrian Rendon**	**Mexico**

Joshua Kim	Australia
David Langton	Australia
Donald Lee	Australia
Matthew Peters	Australia
Lakshman Prassad	Australia
Dimitar Sajkov	Australia
Francis Santiago	Australia
Frederick Graham Simpson	Australia
Sze Tai	Australia
Paul Thomas	Australia
Peter Wark	Australia
**Marcelo Rabahi**	**Brazil**
José Eduardo Delfini Cançado	Brazil
Thúlio Cunha	Brazil
Marina Lima	Brazil
Alexandre Pinto Cardoso	Brazil
**J. Mark FitzGerald** (deceased)	**Canada**
**Andrew McIvor**	**Canada**
Syed Anees	Canada
John Bertley	Canada
Alan Bell	Canada
Amarjit Cheema	Canada
Guy Chouinard	Canada
Michael Csanadi	Canada
Anil Dhar	Canada
Ripple Dhillon	Canada
David Kanawaty	Canada
Allan Kelly	Canada
William Killorn	Canada
Daniel Landry	Canada
Ruth Cerino Javier	Mexico
Alfredo Domínguez Peregrina	Mexico
Marco Fernández Corzo	Mexico
Efraín Montano Gonzalez	Mexico
Alejandra Ramírez-Venegas	Mexico
**Maarten van den Berge**	**Netherlands**
Willem Boersma	Netherlands
R.S. Djamin	Netherlands
Michiel Eijsvogel	Netherlands
Frits Franssen	Netherlands
Martijn Goosens	Netherlands
Lidwien Graat-Verboom	Netherlands
Johannes in 't Veen	Netherlands
Rob Janssen	Netherlands
Kim Kuppens	Netherlands
Mario van de Ven	Netherlands
**Per Bakke**	**Norway**
Ole Petter Brunstad	Norway
Gunnar Einvik	Norway
Kristian Jong Høines	Norway
Alamdar Khusrawi	Norway
Torbjorn Oien	Norway
**Ho Joo Yoon**	**South Korea**
Yoon-Seok Chang	South Korea
Young Joo Cho	South Korea
Yong Il Hwang	South Korea
Woo Jin Kim	South Korea
Young-Il Koh	South Korea
Byung-Jae Lee	South Korea
Kwan-Ho Lee	South Korea

Robert Luton	Canada	Sang-Pyo Lee	South Korea
Piushkumar Mandhane	Canada	Yong Chul Lee	South Korea
Bonavuth Pek	Canada	Seong Yong Lim	South Korea
Robert Petrella	Canada	Kyung Hun Min	South Korea
Daniel Stollery	Canada	Yeon-Mok Oh	South Korea
**Chen Wang**	**China**[a]	Choon-Sik Park	South Korea
Meihua Chen	China[a]	Hae-Sim Park	South Korea
Yan Chen	China[a]	Heung-Woo Park	South Korea
Wei Gu	China[a]	Chin Kook Rhee	South Korea
Kim Ming Christopher Hui	China[a]	Hyoung-Kyu Yoon	South Korea
Manxiang Li	China[a]	**Alvar Agustí García-Navarro**	**Spain**
Shiyue Li	China[a]	**José Olaguibel Rivera**	**Spain**
Ma Lijun	China[a]	Rubén Andújar	Spain
Guangyue Qin	China[a]	Laura Anoro	Spain
Weidong Song	China[a]	María Buendía García	Spain
Wei Tan	China[a]	Paloma Campo Mozo	Spain
Yijun Tang	China[a]	Sergio Campos	Spain
Tan Wang	China[a]	Francisco Casas Maldonado	Spain
Fuqiang Wen	China[a]	Manuel Castilla Martínez	Spain
Feng Wu	China[a]	Carolina Cisneros Serrano	Spain
PingChao Xiang	China[a]	Lorena Comeche Casanova	Spain
Zuke Xiao	China[a]	Dolores Corbacho	Spain
Shengdao Xiong	China[a]	Felix Del Campo Matías	Spain
Jinghua Yang	China[a]	Jose Echave-Sustaeta	Spain
Jingping Yang	China[a]	Gloria Francisco Corral	Spain
Caiqing Zhang	China[a]	Pedro Gamboa Setién	Spain
Min Zhang	China[a]	Marta García Clemente	Spain
Ping Zhang	China[a]	Ignacio García Núñez	Spain
Wei Zhang	China[a]	Jose García Robaina	Spain
Xiaohe Zheng	China[a]	Mercedes García Salmones	Spain

Dan Zhu	China[a]	Jose Maria Marín Trigo	Spain
**Carlos Matiz Bueno**	**Colombia**	Marta Nuñez Fernandez	Spain
Fabio Bolivar Grimaldos	Colombia	Sara Nuñez Palomo	Spain
Alejandra Cañas Arboleda	Colombia	Luis Pérez de Llano	Spain
Dora Molina de Salazar	Colombia	Ana Pueyo Bastida	Spain
**Ulla Weinreich**	**Denmark**	Ana Rañó	Spain
Elisabeth Bendstrup	Denmark	José Rodríguez González-Moro	Spain
Ole Hilberg	Denmark	Albert Roger Reig	Spain
Carsten Kjellerup	Denmark	José Velasco Garrido	Spain
**Pierre-Régis Burgel**	**France**	**Christer Janson**	**Sweden**
**Gilles Devouassoux**	France	Dan Curiac	Sweden
**Chantal Raherison**	France	Cornelia Lif-Tiberg	Sweden
Philippe Bonniaud	France	Anders Luts	Sweden
Olivier Brun	France	Lennart Råhlen	Sweden
Christos Chouaid	France	Stefan Rustscheff	Sweden
Francis Couturaud	France	**Timothy Harrison**	**UK**
Jacques de Blic	France	Frances Adams	UK
Didier Debieuvre	France	Drew Bradman	UK
Dominique Delsart	France	Emma Broughton	UK
Axelle Demaegdt	France	John Cosgrove	UK
Pascal Demoly	France	Patrick Flood-Page	UK
Antoine Deschildre	France	Elizabeth Fuller	UK
Carole Egron	France	David Hartley	UK
Lionel Falchero	France	Keith Hattotuwa	UK
François Goupil	France	Gareth Jones	UK
Romain Kessler	France	Keir Lewis	UK
Pascal Le Roux	France	Lorcan McGarvey	UK
Pascal Mabire	France	Alyn Morice	UK
Guillaume Mahay	France	Preeti Pandya	UK
Stéphanie Martinez	France	Manish Patel	UK

Boris Melloni	France	Kay Roy	UK
Laurent Moreau	France	Ramamurthy Sathyamurthy	UK
Emilie Riviere	France	Swaminathan Thiagarajan	UK
Pauline Roux-Claudé	France	Alice Turner	UK
Michel Soulier	France	Jørgen Vestbo	UK
Guillaume Vignal	France	Wisia Wedzicha	UK
Azzedine Yaici	France	Tom Wilkinson	UK
**Robert Bals**	**Germany**	Pete Wilson	UK
Sven Philip Aries	Germany	**Bradley Chipps**	**USA**
Ekkehard Beck	Germany	Lo'Ay Al-Asadi	USA
Andreas Deimling	Germany	James Anholm	USA
Jan Feimer	Germany	Francis Averill	USA
Vera Grimm-Sachs	Germany	Sandeep Bansal	USA
Gesine Groth	Germany	Alan Baptist	USA
Felix Herth	Germany	Colin Campbell	USA
Gerhard Hoheisel	Germany	Michael A. Campos	USA
Frank Kanniess	Germany	Gretchen Crook	USA
Thomas Lienert	Germany	Samuel DeLeon	USA
Silke Mronga	Germany	Alain Eid	USA
Jörg Reinhardt	Germany	Ellen Epstein	USA
Christian Schlenska	Germany	Stephen Fritz	USA
Christoph Stolpe	Germany	Hoadley Harris	USA
Ishak Teber	Germany	Mitzie Hewitt	USA
Hartmut Timmermann	Germany	Fernando Holguin	USA
Thomas Ulrich	Germany	Golda Hudes	USA
Peter Velling	Germany	Richard Jackson	USA
Sabina Wehgartner-Winkler	Germany	Alan Kaufman	USA
Juergen Welling	Germany	David Kaufman	USA
Ernst-Joachim Winkelmann	Germany	Ari Klapholz	USA
**Alberto Papi**	**Italy**	Harshavardhan Krishna	USA

Carlo Barbetta	Italy	Daria Lee	USA
Fulvio Braido	Italy	Robert Lin	USA
Vittorio Cardaci	Italy	Diego Maselli-Caceres	USA
Enrico Maria Clini	Italy	Vinay Mehta	USA
Maria Teresa Costantino	Italy	James N. Moy	USA
Giuseppina Cuttitta	Italy	Ugo Nwokoro	USA
Mario di Gioacchino	Italy	Purvi Parikh	USA
Alessandro Fois	Italy	Sudhir Parikh	USA
Maria Pia Foschino-Barbaro	Italy	Frank Perrino	USA
Enrico Gammeri	Italy	James Ruhlmann	USA
Riccardo Inchingolo	Italy	Catherine Sassoon	USA
Federico Lavorini	Italy	Russell A. Settipane	USA
Antonio Molino	Italy	Daniel Sousa	USA
Eleonora Nucera	Italy	Peruvemba Sriram	USA
Vincenzo Patella	Italy	Richard Wachs	USA
Alberto Pesci	Italy		
Fabio Ricciardolo	Italy		
Paola Rogliani	Italy		
Riccardo Sarzani	Italy		
Carlo Vancheri	Italy		
Rigoletta Vincenti	Italy		

[a]Data for patients from China were excluded from the present analyses due to a change in regulations about data transfer in May 2019.

National Principal Investigators are shown in bold.

FIGURE LEGENDS

Figure S1. Number of patients categorized as pre-COPD, PRISm, GOLD 1-2 or GOLD 3-4 at recruitment according to a fixed FEV1/FVC value < 0.7 (X axis) that would be re-classified if an

FEV1/FVC< its Lower Limit of Normal (LLN) would have been used instead (Y axis). For further explanations, see text.

Figure S2. Forest plots showing the adjusted Odds Ratios (OR) comparing several demographic and exposure variables in pre-COPD and PRISm patients. For further explanations, see text.

Figure S3. Forest plots showing the adjusted Odds Ratios (OR) comparing several symptoms (Panel A), disease characteristics (Panel B), exacerbations (Panel C) or comorbidities (Panel D) in pre-COPD and PRISm patients. For further explanations, see text.

Figure S4. Forest plots showing the adjusted Odds Ratios (OR) comparing several biomarkers in pre-COPD and PRISm patients. For further explanations, see text.
