# Supplementary figures and images for: The many faces of COPD in real life: a longitudinal analysis of the NOVELTY cohort
Source: ERJ Open Res. 2024 Feb 12;10(1):00895-2023. doi: 10.1183/23120541.00895-2023 (PMC10860203; doi:10.1183/23120541.00895-2023)

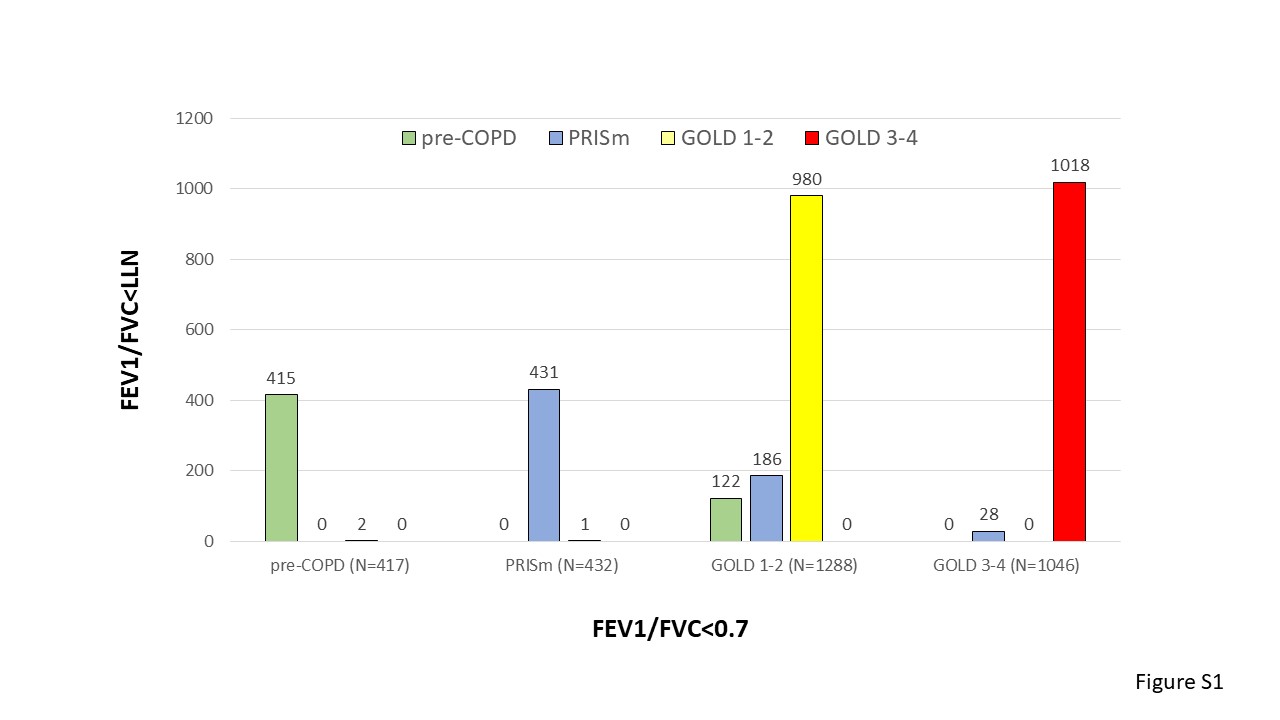

Supplement: Supplementary file 2 [file 00895-2023.FIGURES1.jpg]

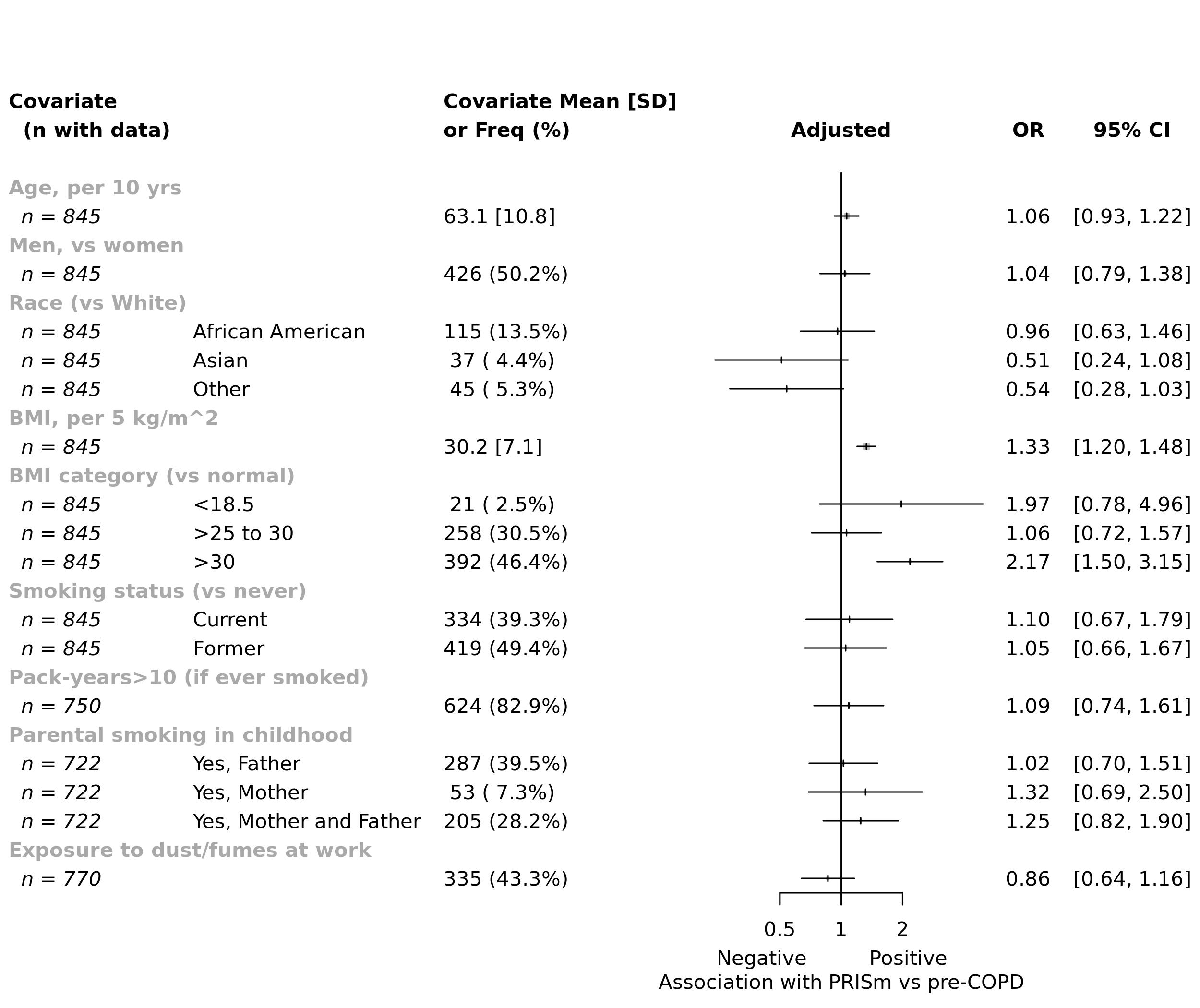

Supplement: Supplementary file 3 [file 00895-2023.FIGURES2.jpeg]

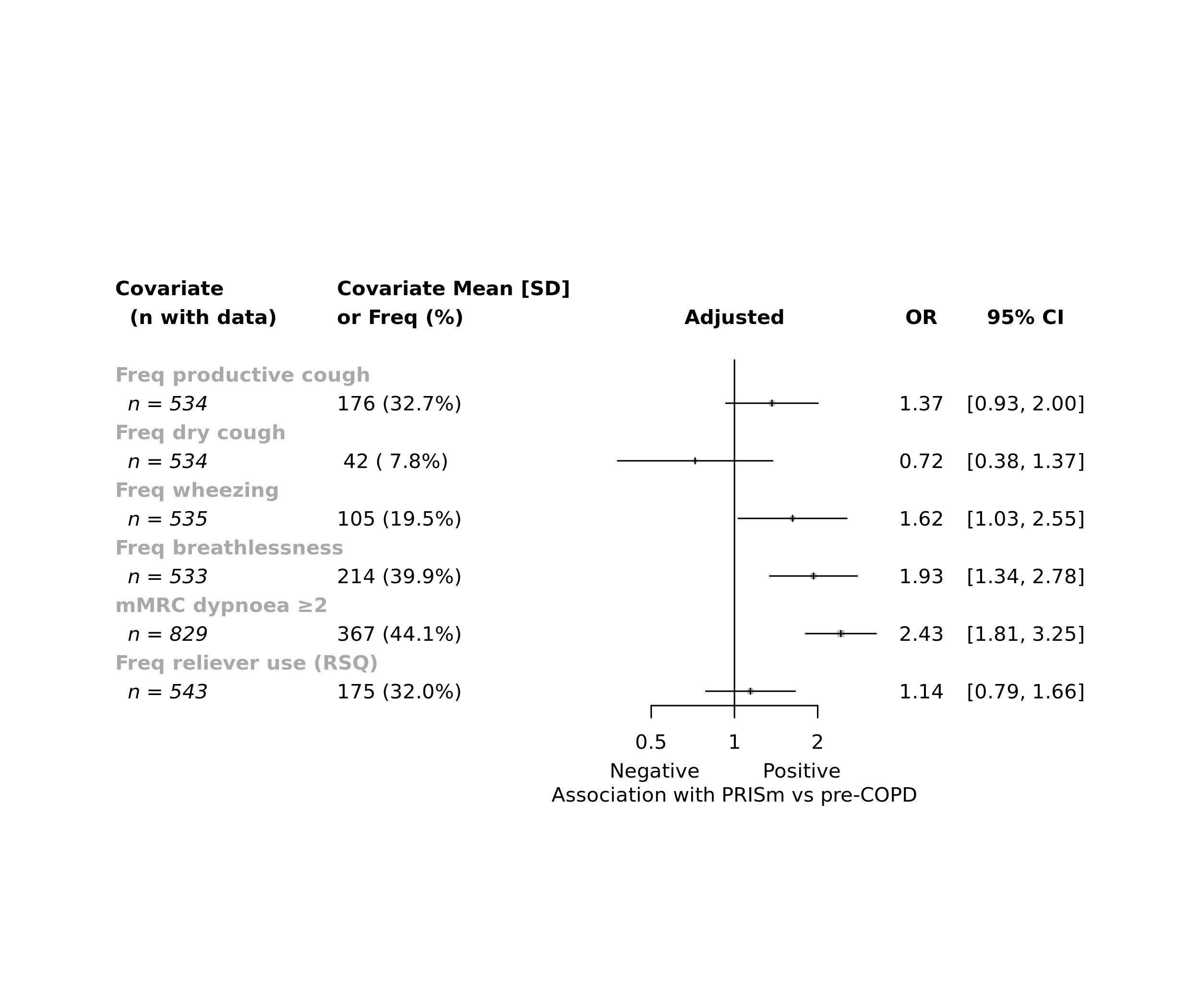

Supplement: Supplementary file 4 [file 00895-2023.FIGURES3A.jpeg]

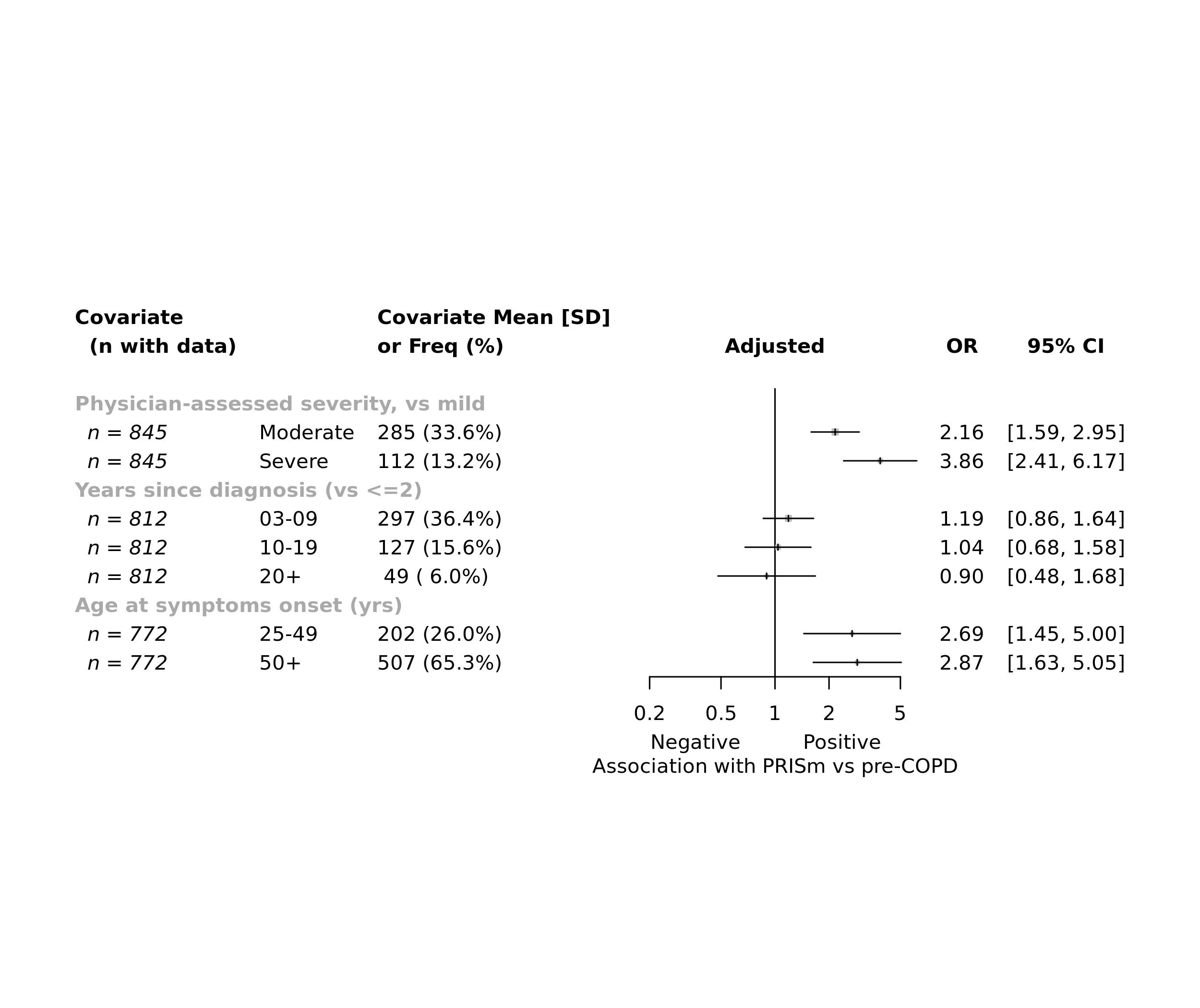

Supplement: Supplementary file 5 [file 00895-2023.FIGURES3B.jpeg]

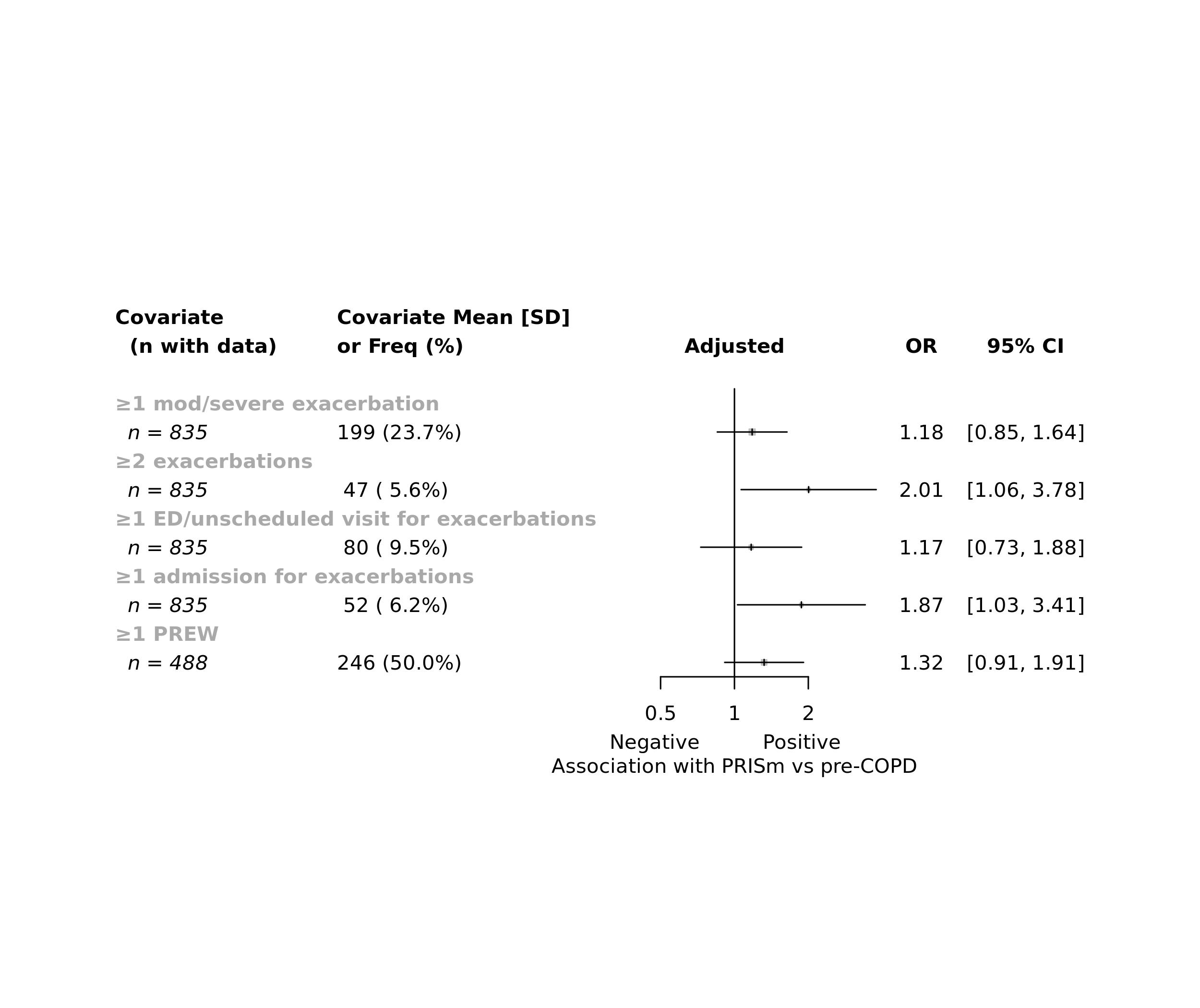

Supplement: Supplementary file 6 [file 00895-2023.FIGURES3C.jpeg]

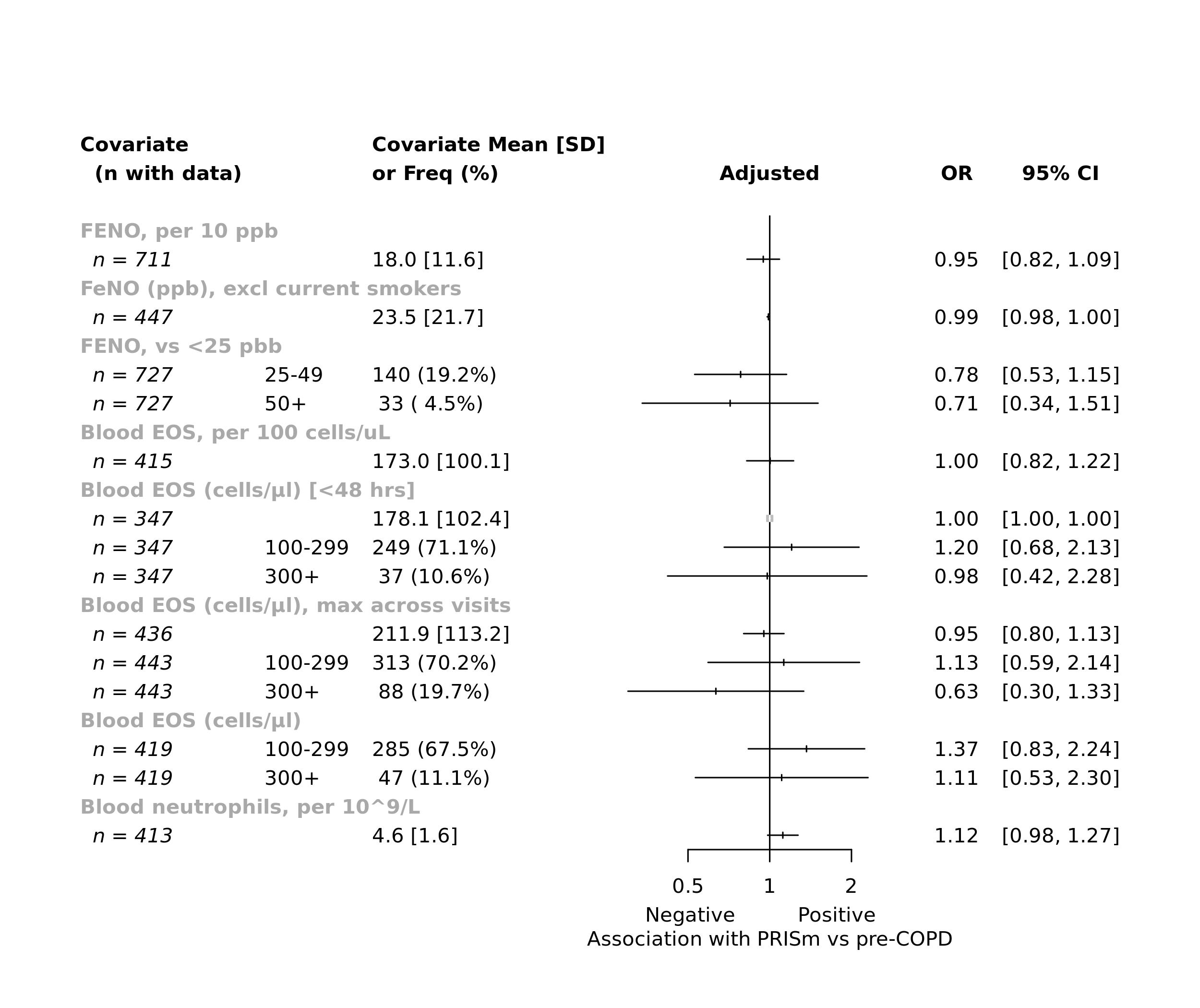

Supplement: Supplementary file 7 [file 00895-2023.FIGURES4.jpeg]
